# Silica/Protein and Silica/Polysaccharide Interactions and Their Contributions to the Functional Properties of Derived Hybrid Wound Dressing Hydrogels

**DOI:** 10.1155/2021/6857204

**Published:** 2021-11-03

**Authors:** Monica Mesa, Natalia Y. Becerra

**Affiliations:** ^1^Materials Science Group, Institute of Chemistry, University of Antioquia, Medellín 050010, Colombia; ^2^Tissue Engineering and Cell Therapy Group, Faculty of Medicine, University of Antioquia, Medellín 050010, Colombia

## Abstract

Multifunctional and biocompatible hydrogels are on the focus of wound healing treatments. Protein and polysaccharides silica hybrids are interesting wound dressing alternatives. The objective of this review is to answer questions such as why silica for wound dressings reinforcement? What are the roles and contributions of silane precursors and silica on the functional properties of hydrogel wound dressings? The effects of tailoring the porous, morphological, and chemical characteristics of synthetic silicas on the bioactivity of hybrid wound dressings hydrogels are explored in the first part of the review. This is followed by a commented review of the mechanisms of silica/protein and silica/polysaccharide interactions and their impact on the barrier, scaffold, and delivery matrix functions of the derived hydrogels. Such information has important consequences for wound healing and paves the way to multidisciplinary researches on the production, processing, and biomedical application of this kind of hybrid materials.

## 1. Introduction: Key Definitions and Analysis Techniques Related to Silica and Hydrogels

**Figure 1:**
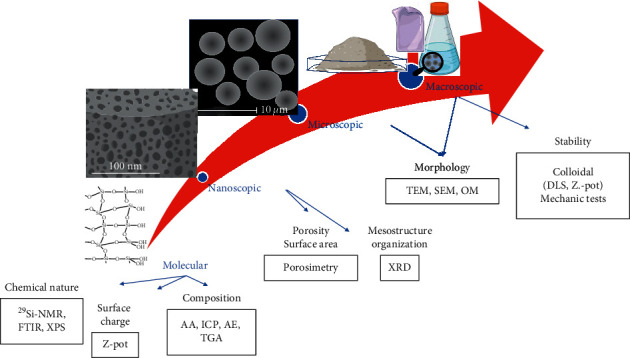
Silica properties and common characterization techniques at different levels.

Silica is a silicon oxide inorganic network, bearing a low population of silanol groups on the surface. This material has a zero-point charge ∼ pH 2.2–3.0. The geminal and single silanol groups have pKa around 5.6 and 10.5, respectively [1]. Silica can be synthesized by bottom-up approximations from common precursors such as alkoxysilanes (tetraethylortosilane, TEOS; tetramethylortosilane, TMOS), organosilanes also known as organoalkoxysilanes or alkylalkoxysilanes (3-glycidoxypropyltrimethoxysilane, GPTMS; 3-aminopropyltriethoxysilane, APTES), and/or sodium silicate [2]. The morphology, porosity, specific surface area, and chemical composition can be modulated by selecting the synthesis conditions and postsynthesis modifications [3]. Some usual characterization techniques are summarized in Figure 1.

Silica is usually present in the form of films, colloidal suspensions, and powders of particles and fibers. The sodium silicate and Ludox® are commercial examples of colloidal suspensions, which have between 30 and 50 wt% SiO_2_, nonporous nanoparticles (above 25 nm), and Na^+^ or NH_4_^+^ counterions in a basic aqueous medium. The charge of the deprotonated silanol groups in this medium is responsible for the electrostatic colloidal stability [4]. The temperature increasing and/or pH lowering favor the interparticle silanol condensation, leading to particle aggregation/growing. These groups behave as primary alcohols (-Si-OH), and their condensation results in -Si-O-Si- and -Si-O-R siloxane networks (analogous to organic esters, where *M* can be a metal or carbon). This reaction is responsible for the silica roles as binder and promoter of adhesion [5]. Moreover, the same reactions are also involved in the silica postfunctionalization with organic groups by silanization or silylation reactions with organoalkoxysilanes and chlorosilanes.

Frequently, the silica particles are added as reinforcers to polymers for favoring the hardness, microbial, and attrition resistance of the resultant composites or hybrid materials. This has motivated their use in hydrogels for production of wound dressings, as reported for similar natural clays and other related metal oxides [6–10]. The impact of these properties will be exemplified on the subsequent sections of this review. Moreover, the silica has osteogenic and angiogenic effects that are interesting for tissue engineering [11, 12].

Especial emphasis has been put on the production and properties characterization of hydrogel wound dressings derived from proteins (fibrin, collagen, elastin, gelatin, silk fibroin, and keratin) [13, 14] and polysaccharides (cellulose, chitosan, chitin, pullulan, starch, and *β*-glucan) [7, 15]. These kinds of hydrogels could mimic tissues and could provide an adequate environment for tissue regeneration, due to their physicochemical and biological characteristics. These hydrogels can adsorb high quantities of water and can interact with extracellular matrix moieties to drive cells–hydrogels interactions through the presence of hydrophilic groups (e.g., carboxyl, hydroxyl, ether, and amino groups).

The microstructure of both types of hydrogels relies on the physical and/or chemical crosslinking [16], which in turn affect their physicochemical properties [17]. The wound dressing functions are also directly affected by the crosslinking degree. Physicochemical properties related to the crosslinking such as the mechanical stability of these hydrogels can determine their use so that they can be properly manipulated [18]. Their morphology can make these hydrogels efficient as cell culture supports and favor growth, proliferation, and even differentiation of cells that allow tissue regeneration [19, 20]. Other structural properties such as swelling ratio, glass transition, and protein denaturation could determine the efficient nutrient transport and cellular interactions (adhesion) inside these hydrogels, which make them good candidates for wound dressings. A quick guide for crosslinking related properties characterization is given in Table 1.

Then, the question that addresses this review is how silica properties and its physicochemical interactions with proteins or polysaccharides affect the mechanical, chemical, and biological properties of the derived wound dressing hybrid hydrogels. The objective is to explain the contributions of tailoring the porous, morphological, and chemical characteristics of synthetic silicas on the bioactivity of hybrid wound dressings. This is followed by a critical review of the mechanisms of silica/protein and silica/polysaccharide interactions and their impact on the barrier, scaffold, and delivery matrix functions of the derived hydrogels, which are important for wound healing.

The novelty, originality, and relevance of the review rely on the following aspects.

- This review explains how to tailor silica properties to enhance bioactivity in wound healing processes and encourage the use of siliceous materials in wound dressings.
- This is the first review that focuses on the mechanisms of silica/protein and silica/polysaccharide interactions involved in the physical entrapped or covalent bonding of preformed silicas or their silane precursors onto hydrogels and explains their functional properties as wound dressings
- This review widens the basic knowledge for designing hybrid wound dressings based on silica, protein, and polysaccharides hydrogels, starting from the tailoring of silica chemistry and modulating the molecular interactions during the synthesis and gelation processes, respectively.
- This review rationalizes the impact of silane and silica on the functional properties of protein and polysaccharides derived hydrogel from a chemical point of view.

## 2. Relationships between Silica Properties and Wound Healing Bioactivity

Nowadays, research focuses on the production of wound dressings that promote cellular migration/adhesion/proliferation, hemostasis, and tissue regeneration in conjunction with the passive task (barrier) of the gauzes. Recent reviews on wound healing highlight the participation of natural clays, minerals, and related materials in different stages of this biological process [10] and their use for the preparation of multifunctional bioactive inorganic/organic nanocomposites by different methods [7, 9]. Specifically, silicas synthesized in the laboratory become versatile materials because their chemical, porous, and morphological properties can be tailored according to the application, as it has been reported for multiple biomedical areas [3].

**Figure 2:**
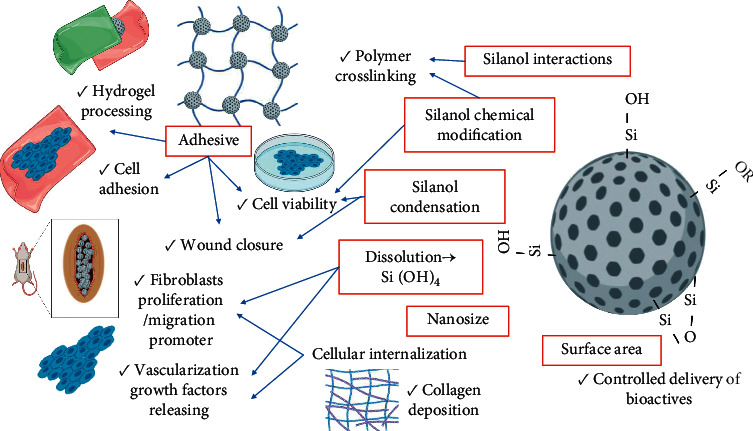
Silica properties (red squares) and biological responses on wound healing and wound dressings production.

The next examples show that silica *per se* contributes to the bioactivity of wound dressings, emphasizing how their physicochemical properties affect the biological responses on different in vivo and in vitro experiments (Figure 2).

### 2.1. Specific Surface Area, Porosity, and Morphology

The surfactant-templated sol-gel or miniemulsion methodologies can be adapted for producing colloidal silicas with different particle (30–75 nm) and pore (2.7–6.5 nm) sizes. They were stronger polymer gel adhesive than nonporous silica (LUDOX TM-50). The larger nanoparticles and higher pore diameters increased the polymer contact due to their higher total outer surface area (513.61 m^2^/g). The silanol groups can be interacting with the polymers or cells mainly through H-bonds and electrostatic forces, increasing the contact area between these surfaces. Moreover, the silica condensation under dehydration conditions or pH changes promotes the formation a silica network that eventually could also help to join the two surfaces, as it has been shown in other materials [24]. Therefore, the silica nanoparticles act as glue for biological tissues [25]. This helps to explain their adhesive role for wound closure when present in chitosan-derived hydrogels [5]. The silica nanoparticles also favor the collagen deposition without the risk of accumulation. The later is due to slow degradation in a physiological medium [26]. The high porosity of the silica is also responsible for the enhancement of the adhesion and proliferation of fibroblast cells in the drug-loaded chitosan–silica/poly(*ε*-caprolactone) wound dressing [27]. The porosity contributed to the adsorption and sustained release of keratinocyte growth factor from a hybrid chitosan-silica dressing, accelerating wound healing [28]. An increase of the surface area, due to the silica nanospheres on chitosan materials, was responsible for improved bleeding homeostasis. Silica promoted blood adsorption and platelet aggregation [29, 30].

### 2.2. Surface Charge

Undersaturated suspensions of silica (below 2 mM Si), prepared from sodium silicate, with primary particles ∼ 10 nm normally exhibit negative charge at basic pHs. The negative character can be increased by the presence of L-(+)-lysine in the reaction mixture or be changed for being positive by postfunctionalization with APTES. Their use in an in vitro study showed that the positively charged nanoparticles stimulated the human skin fibroblasts (CCD-25SK) proliferation/migration and wound closure. This is because of the easy cellular internalization and controlled intracellular dissolution, releasing the beneficial silicic acid and maintaining the cell viability [31]. The silicic acid (1 ug/mL Si^+4^, extracted from a commercial bioglass) is not cytotoxic for human umbilical venous endothelial cells (CCK-8 assay) and promotes the release of vascularization growth factors (HIF-1*α*, VEGF) [32].

### 2.3. Chemical Modifications

A high concentration of silanol groups would make the silica surface reactive towards the blood cells and can promote the reactive oxygen species formation. Therefore, the control of the chemical nature of the surface is important for avoiding hemolysis, especially if they are going to be used on wound dressings when the dermis is compromised. The silanol condensation by thermal treatments and the coating of nanoparticles with organic molecules are some strategies for avoiding this effect [33]. For example, the covalent attachment of chitosan through the isocyanatopropyltriethoxysilane linker and subsequent grafting of hydrocinnamic acid mediated by N-(3-dimethylaminopropyl)-N′-ethylcarbodiimide hydrochloride (EDC) reactant avoid the concentration-dependent cytotoxicity of silica nanoparticles. They improve also the clotting kinetics on whole blood and in vivo hemostasis because the chitosan promotes the amine-blood cells by electrostatic interactions and the catechol quinones can be bound covalently to carbohydrates and proteins (thiol, amine, and imidazole residues) of extracellular matrix, creating a physical barrier by accumulation [34]. The silica particles can be also marked with fluorophores for having smart wound dressings able to sensing bacterial infections [35] and follow the in vivo permeability and cellular uptake [30, 36, 37].

In addition to the bioactive effects of silicas, the inorganic nature, tailored surface area, and loading capacity favor their application as polymers' reinforcing agent and drug/cell carrier [3, 38, 39]. These aspects have also positive impacts on the mechanical properties (for the processing, handling, etc.) and biological behavior of the hydrogels, as will be explained in the following section.

## 3. Mechanisms of Silica/Protein and Silica/Polysaccharide Interactions and Their Impacts on the Functional Properties of the Derived Hydrogels

**Figure 3:**
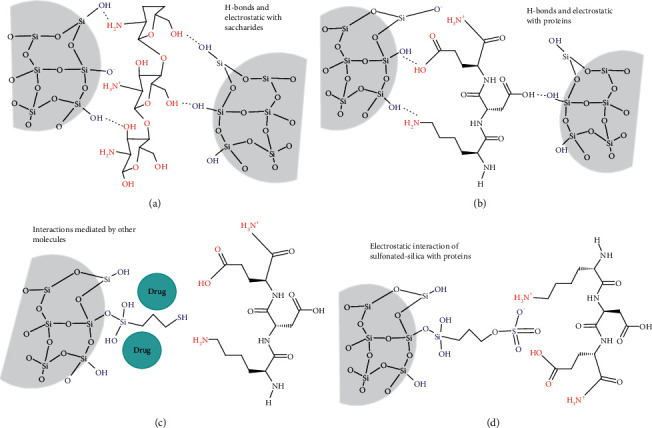
Physical interactions of pure (a,b) and silylated silicas (c,d) with polysaccharides and proteins by direct or mediated mechanisms.

**Figure 4:**
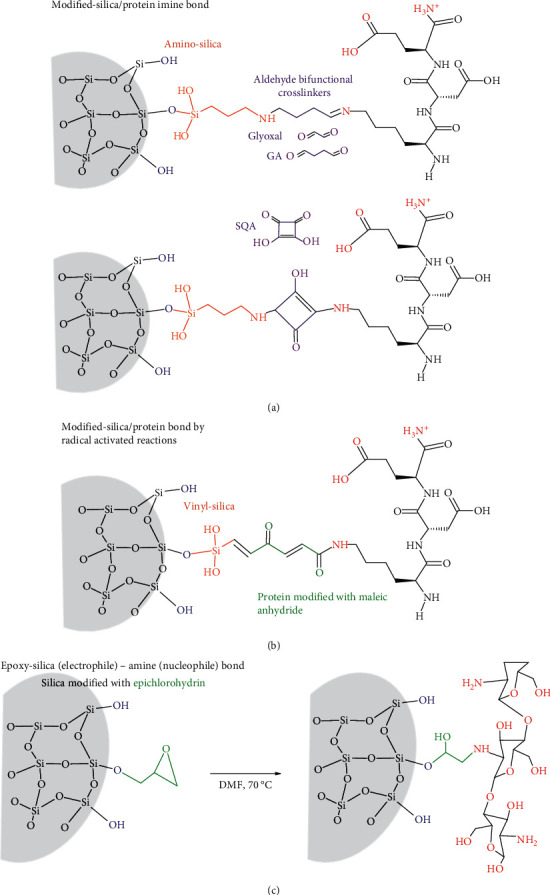
Chemical modification of silica and their contributions to their covalent bonding to proteins through (a) aminosilica-protein imine bonds mediated by bifunctional aldehyde crosslinkers (glyoxal; GA: glutaraldehyde; SQA: squarid acid); (b) radical activated reactions between vinyl-silica and maleic anhydride protein; (c) polysaccharide coupling via the epoxy opening in silica modified with epichlorohydrin at 70°C in dimethylformamide (DMF).

**Figure 5:**
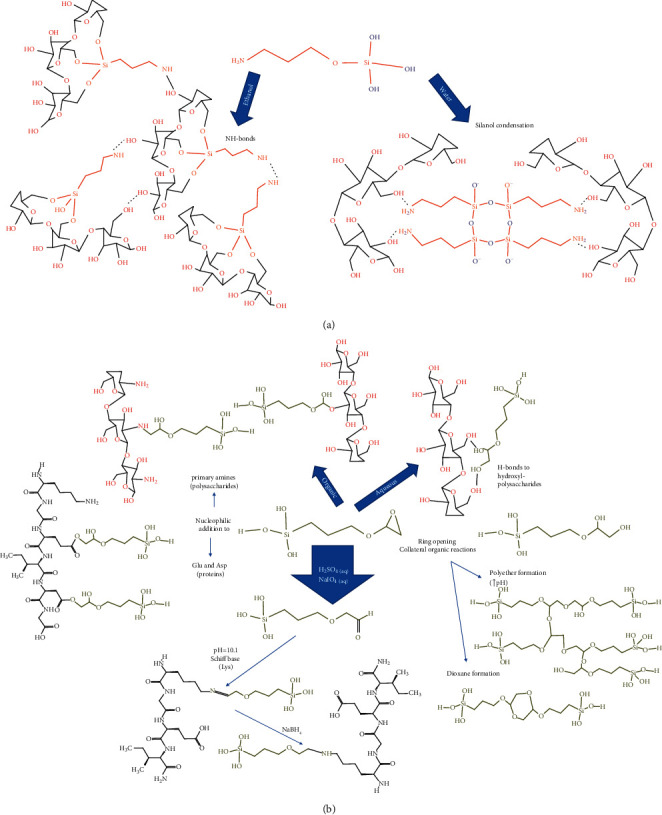
Multiple reactions and interaction mechanism of (a) APTES and (b) GPTMS silanes in the presence of proteins and polysaccharide hydrogel precursors.

The silica and its sources can be incorporated by physical entrapment or covalent bonding onto hydrogels (Figures 3–5). This is independent if the gels are prepared by three-dimensional polymerization of monomers, cross-linking of water-soluble polymers [16, 40], or self-assembly of proteins mediated by different kinds of molecular interactions and external stimulus [41, 42]. The next discussion will be focused on the interaction mechanisms and multiple contributions of silica to the bioactivity and passive action (barrier and selective permeability) of polysaccharides- and protein-derived hydrogels, including injectable and molded wound dressings and scaffolds.

### 3.1. Physical Entrapment

The silica-hydrogels composites mediated by physical interactions can be prepared by mixing the protein or gel precursor with the (i) silica source or (ii) preformed silica nanoparticles (Figure 3).

#### 3.1.1. Gelation in the Presence of the Silica Source

An example of route (i) is the foam membrane prepared by lyophilization of TMOS and chitosan colloidal dispersions, giving a composite foam [43]. The TMOS silanol groups (pKa > 5.6) interact with the amine (pKa = 6.3–6.5) and hydroxyl groups of this polysaccharide through electrostatic and Van der Waals/hydrogen bonds interactions during the gel formation (Figure 3(a)), as demonstrated for similar systems at neutral pH [44]. These interactions can reduce the polymer mobility, which explains why silica reinforces the gel, helping to maintain the foam structure after adsorption of larger wound exudate volumes compared with the pure chitosan gel [43, 45]. This water adsorption capability and faster wettability are induced by the silica surface hydrophilicity, which is also important for cell viability. The in vivo tests show that the silica presence enhances the fibroblast proliferation, secretion of TGF-*β* growth factor, and collagen deposition [43].

The chemical nature, the concentration of the silica source, and temperature affect the protein autoassembly, which has consequences on the hydrogel microstructure. For example, the APTES polycondensation during gelation of fibrinogen from bovine serum produced reinforced fibrin hydrogels with more homogenous fiber diameters than TEOS at lower concentrations, without affecting the myoblast growing in both cases. The differences were explained by the ammonium functionality APTES, which contribute to the electrostatic interactions with proteins and modulate the ionic strength of the medium, affecting the fiber thickness and stiffness [46]. On the other hand, the increase of the temperature above the denaturation protein (70°C) can be responsible of changes on the fibrinogen association and interaction strength with anionic silicate species (from sodium silicate) by the Arg and Lys cationic rich region at pH 3. They affect the stiffness of the fibrin hydrogel and the silica condensation extent and microstructure [47].

#### 3.1.2. Gelation in the Presence of the Preformed Silica Materials

The route (ii) involves the use of preformed pure or functionalized silica, having also physical and biological contributions. The hydrogen bond interactions between silica nanoparticles and glycol chitosan restricted the movement of polymer chains, which affected the sol-gel transition, elastic (G′), and loss (G″) moduli and improved the adhesive properties of this injectable hydrogel [5]. These particles can be nucleation agents for hydrogels polymerization and/or crosslinking. For example, the presence of pure silica on the blood plasma gelation induced by CaCl_2_ and crosslinking with glutaraldehyde increase the fiber thickness and hydrogel porosity [48]. This is due to their interaction with fibrinogen (isoelectric point = 5.5) especially through the electrostatic interactions (NH^3+^ vs. SiO-) in the *α*-domain, as studied by sodium silicate polycondensation in the presence of fibrinogen [47]. Other Van der Waals interactions and H-bonds can be involved (-NH and -OH vs. Si-OH and SiO-) (Figure 3(b)), usually in protein-derived hydrogels depending on the pH of the medium and pKa of the amino acids [49]. The size and concentration of silica nanoparticle nuclei must be controlled for maintaining the fibrillar organization and avoid silica aggregation. This is because nanoparticles are reinforcing charges if they are homogeneously distributed through the gel [50]. On the contrary, the aggregates can be breakdown points under mechanical stress, as exemplified with elastic collagen hydrogels [51]. Silica also increases the elastic behavior of the injectable cellulose and chitosan hydrogels [5, 52] and the tensile strength of silica-cellulose membranes [53]. Another physical contribution of silica nanoparticles is related to the swelling. It is demonstrated how the highly organized porous structure of SBA-15 mesoporous silica added during the alkaline casting and acidic recovering of cellulose films improves the swelling rate, conferring a 1012 to 1186 g·m^−2^·d^−1^ moisture permeability. This is into the ideal values (904–1447 g·m^−2^·d^−1^) for wound dressings [53].

The nanoparticles can preserve the biological contributions discussed in the precedent section after their incorporation on the hydrogels. This is because their dissolution under physiological conditions produces silicic acid [26, 32], which can be progressively delivered from the hydrogel. For example, from fibrin scaffolds without affecting the cell behavior [48] and showing wound healing synergic effects on succinyl chitosan/dialdehyde-based oxidized alginate crosslinked injectable hydrogel, favoring collagen/myofibrils accumulation, new blood vessels formation, and L929 fibroblast viability [52]. On the other hand, silica can also be a reservoir of bioactive compounds, such as keratin growth factors and interleukins, improving and expanding the biological functions of the wound dressings [28, 54].

A special interest has been put in the controlled release of antimicrobial agents. They can be inorganic oxides such as ZnO incorporated during the synthesis of bioactive glass nanoparticles [52], Cu nanoparticles covered by silica in a starch medium [55], or organic antibiotics adsorbed on pure [51, 56, 57] or chemically-modified [39, 58–60] nanosilicas, before being added to the gel. Silica microspheres loaded with ferulic acid during TEOS polymerization conferred antimicrobial and antioxidant properties to fibrin/chitosan/keratin wound scaffolds [61]. The delivery profile and cytotoxicity are dependent of particle size/concentration, as exemplified with collagen hydrogels [51]. Moreover, the silica chemical modification can modulate the hydrophobicity and charge of the surface, which in turn affect the antibiotic diffusion from the wound dressing. This was the objective of silica functionalization with (3-mercaptopropyl) trimethoxysilane for having hydrophobic propyl-thiol groups (Figure 3(c)) and treatment with sulfuric acid for having negative charge (sulfonate; Figure 3(d)) groups [58]. These chemical modifications could affect also silica interactions with protein precursor; nevertheless, they continue being physical (Figure 3(c)) because the abundance of nucleophilic cysteines (pka 8) is so low and there are not oxidant agents promoting a disulfide formation [62]. On the other hand, the delivery can be also driven by the silica dissolution and hydrogel degradation. This was shown with preloaded FICT-lysozyme silica supraparticles assembly, coated with fibrin polymerized from fibrinogen in the presence of thrombin [63].

Recently, *γ*-chloropropyl-triethoxysilane (CPTES) was used as the cross-linking agent between N-halamine chemically modified silica particles and chitosan. The silanol groups of the poly(CPTES) chains, formed at 80°C, interact with the amine and hydroxyls groups of the chitosan. This physical entanglement leads to a stable, low density, 3D interconnected porous structure with additional swelling, hemostatic, and bactericidal properties [64].

### 3.2. Covalent Bonding

The silica can contribute to the covalent bonding of hydrogels in form of (i) silylated silicas, whose surface chemical group will act as coupling and crosslinker functionalities, and (ii) silsesquioxanes and oligomers formed after coupling an alkoxysilane to the protein or polysaccharide hydrogel precursor (Figure 4).

#### 3.2.1. Functionalization of Silicas for Their Use in Hydrogels

Silica particles or fibers can be chemically modified by condensation their silanol groups with organoalkoxysilanes, chlorosilanes, or organochlorines. The APTES is a common organosilane [65] for coupling the silica to bifunctional crosslinkers such as glutaraldehyde, glyoxal [66, 67], and squaric acid [68], typical in the production of protein-derived hydrogels (Figure 4(a)). The silanization process allows controlling the amine density, nucleophilicity, and ionization degree for being physical and covalent reactive attachment multipoints in the silica surface [69]. The covalent coupling with glutaraldehyde occurs at a pH higher than 7.6, which is the pka of the amino groups in this kind of amino-silica surface [70]. The modification degree can be measured indirectly by the free-amino groups by the OPA method [67]. After that, the aldehyde groups on the chemically modified silica particles condensate mainly with the primary amine groups of lysines (pka 10.3), forming an imine bond (Figure 4(a)). For example, they lead to a highly dense chemical gelatin crosslinked microstructure with a more elastic behavior and lower swelling volume and water desorption at a lower temperature than the gel prepared in the presence of nonmodified silicas [49]. The silica functionalized with APTES/glutaraldehyde in the form of films can also support the formation of smooth and homogenous protein monolayer. That was the case of a keratin scaffold, promoting the adhesion and proliferation of human dermal fibroblasts and epidermal keratinocytes [71]. Another crosslinker is the genipin (Figure 4(a)), whose proposed crosslinking mechanism involves the nucleophilic attack of primary amines of the protein and dimerization [72], being a second-order reaction on fibrin hydrogels [73]. The chemical crosslinking mediated genipin-amino-silicas avoided the inorganic/organic phase separation due to nanoparticles aggregation in collagen/chitosan/hyaluronic hydrogels, increasing the hydrogel stiffness [74].

Even less common, the silica nanoparticles modified vinyltrimethoxysilane can be also reactive crosslinkers (Figure 4(b)). Their vinyl groups participate in radical activated reactions with a maleic anhydride modified-casein and glycidyl methacrylate modified-chondroitin, started by sodium persulfate in N,N,N′,N′-tetramethyl ethylenediamine and ended by combination or disproportion reactions. This gave a chemical crosslinked gel, where the silica nanospheres were well-dispersed in protein-derived hydrogels, and controlled the sustained release of the selected drug, as they interfere with the interactions between the drug and the protein matrix [75].

Organochlorine such as epichlorohydrin can be also used for the chemical modification of silica. It reacts with the free silanol, forming a carbon-oxygen bond and releasing HCl in organic solvents at 70°C. The epoxy functionality on these functionalized particles is an electrophile, which can react with amine groups in saccharides such as chitosan (Figure 4(c)), via the epoxy opening pathway, contributing to the polymer crosslinking. The hybrid nature has synergic effects on the wound healing, exudates adsorption, and gas exchange of the wound dressings [76].

#### 3.2.2. Functionalization of Protein or Polysaccharide Hydrogel Precursor with Silanes

The coupling of organoalkoxysilanes to the protein or saccharide hydrogel precursor can occur by physical and covalent interactions (Figure 5). These organoalkoxysilanes behave as bifunctional reagents, whose chemical grafting and the extent of organic and inorganic polymerization depend on the reaction conditions. For example, the chemical grafting of APTES through the silanol groups to cellulose nanofibrils is favored in ethanol and drying at 105^o^C; by the contrary, the reaction in water at room temperature promotes the H-bonds interactions between the primary amine of the alkylalkoxysilane with the hydroxyl of the polysaccharide [77]. Then, the selection of the solvent and temperature is the key for having a free amine or silanol groups, contributing to the chemical crosslinking of the hydrogel [77] through the H-bonds or siloxane formation (Figure 5(a)). The amine behaves as a basic catalyst, promoting particle aggregation [78]. Therefore, special care must be put in controlling its concentration for avoiding the formation of breakdown clusters that affect the hydrogel mechanical properties.

The GPTMS is another alkoxysilane, in which the two electrophilic carbons on the epoxy ring are prone to the nucleophilic attack of primary amine groups on polysaccharides and proteins, forming secondary amines (Figure 5(b)). However, the chemical grafting depends on the polymer type, reactants concentration, pH, temperature, and addition of silica [79]. In the case of silica-chitosan scaffolds, the ^13^C and ^29^Si NMR show the nucleophilic addition is through the primary amine of chitosan pH 2–4 and 40°C, accompanied by the epoxy ring-opening and diol formation. The condensation degree of the free silanol groups depends on the pH and the addition of hydrolyzed TEOS, which in turn affect the dissolution, porosity, and mechanical properties of the scaffolds form by freezing casting [80]. The increasing of GPTMS concentration improves the chemical grafting and crosslinking degree on the chitosan composites (followed by FTIR and ^29^Si-NMR), decreasing the porosity and retarding the scaffold dissolution [81].

The probability that the glyoxyl group could be covalently attached to the hydroxyl and silanol groups is lower compared with the amines, taking into account that the reaction rates towards epoxy groups follow the tendency RSH > RNH_2_, R_2_NH > RCOOH > SiOH » ROH > H_2_O [82]. Therefore, the H-bonds interactions occur with the diols formed under acid conditions [82], contributing also to the polysaccharides-derived hydrogel crosslinking (Figure 5(b)). On the other hand, reactions in organic solvents [83] allow the glyoxyl chemical modification of hydroxyl-reach polysaccharides. For example, the further free silanol groups' condensation with silica nanofibers in water improves the mechanical properties and water diffusion of silanized hydroxypropylmethylcellulose hydrogel [84, 85].

The GPTMS for protein chemical modification occurs mainly through the glutamic and aspartic amino acids instead of lysines and arginines (Figure 5(b)). This is because acid residues are the most abundant and better nucleophiles at pH between 5 and 7, where this protein modification is commonly made. That was the case of gelatin-derived hydrogels, where the epoxy reactions and the condensation of the silane also occur simultaneously in the presence of the Bronsted catalyst at pH 5, producing a gel when the reaction time is long and temperature is higher than 40°C [86]. This kind of scaffolds is biocompatible, and the porosity and mechanical strength can be controlled by the inorganic–organic coupling for being scaffolds on tissue engineering [87, 88]. Moreover, the chemical crosslinking increases the hydrophobicity and thermal stability of these scaffolds [89]. The GPTMS hydrolysis at highly acid pH (H_2_SO_4_ aqueous solution) and oxidation with NaIO_4_ transform the epoxy into an aldehyde functionality, which forms a Schiff base with lysine residues at pH higher 10.1 (pK of the Lys). Then, it can be stabilized by reduction with sodium borohydride (Figure 5(b)). At basic pH, the kinetically controlled inorganic condensation of 3-glycidoxypropyltrimethoxysilane predominates, affecting the opening and organic reactions of the epoxy ring. The sol is stable for months [90]. The ring-opening and organic reactions are more efficient when the NaOH is increased [91] and can occur also in the polyether formation (Figure 5(b)). Then, self-reactions and interactions with proteins and polysaccharides are competing. Therefore, the selection of the medium conditions must be carefully chosen for promoting the hydrogel crosslinking.

## 4. Concluding Remarks and Perspectives

The silanes and silicas contribute to the barrier, scaffold, and delivery matrix functionalities of wound dressings hydrogels. They modify the physicochemical and biological characteristics of the hydrogel. The adhesive properties of these inorganic compounds by silanol condensation can be useful for the hydrogel formation and cells/hydrogels interactions. Physical and covalent mechanisms of silane and silica interactions with protein and polysaccharides could modulate the crosslinking degree and bioactivity of the derived hydrogels. The former has consequences on the elasticity and stiffness mechanical properties, wetting, permeability (nutrient transport), and degradation of the hybrid hydrogel. Moreover, silica has positive effects on the bioactivity of the wound dressing, directly through the dissolution up to silicic acid or by carrying bioactive molecules (growth factors, antioxidants, and antibiotics).

The reinforcement of polysaccharide and protein derived-hydrogels for having multifunctional wound dressings is an open interdisciplinary field of research. The chemical modification of silica with crosslinkers, markers, and bioactive molecules gives opportunities of having first, second, and third-generation hydrogels (analogs to the hierarchical nanocarriers classification), which is beneficial to produce smart materials. The high relevance of silica on the mechanical properties of these natural hydrogels encourages the research of new processing and characterization methods for increasing the yield efficiency and sensible testing. The medical staff and users have more alternatives to conventional wound healing treatments.

## Figures and Tables

**Table 1:** Protein and polysaccharide derivates hydrogels crosslinking related properties characterization.

Hydrogels source	Measurement method	Crosslinking related property	References
Polysaccharide: alginate	Compressive test	Stiffness (elastic modulus)	[20]
Polysaccharide: alginate Protein/polysaccharides collagen/tyramine hyaluronan	Gravimetric analysis	Degradation	[18, 19]
Polysaccharide: alginate	DNA content Metabolic assay test	Cell proliferation	[17–21]
Proteins and polysaccharides: alginate, collagens, fibrin, hyaluronic acid, keratin, and laminin	Atomic force microscopy AFM	Stiffness (elastic modulus) Microstructure	[18, 21, 22]
Protein/polysaccharides collagen/tyramine hyaluronan Fibrin/alginate	Rheological measurements	Storage *G*′ and loss *G*″ moduli	[19, 22]
Polysaccharide: inulin Protein/polysaccharides collagen/tyramine hyaluronan	Differential scanning calorimetry DSC	Glass transition Protein denaturation	[19, 23]
Polysaccharides: alginate; inulin Protein: fibrin Protein/polysaccharides collagen/tyramine hyaluronan	Scanning electron microscopy (SEM)	Porous diameter; morphology	[18, 19, 23]
Polysaccharides: inulin Protein/polysaccharides collagen/tyramine hyaluronan	Gravimetric analysis	Swelling ratio	[19, 23]

## Data Availability

The data used to support the findings of this study are available from the corresponding author upon request.
